# Comparison of nuclear imaging techniques and volumetric imaging for the prediction of postoperative mortality and liver failure in patients undergoing localized liver-directed treatments: a systematic review

**DOI:** 10.1186/s13550-021-00816-4

**Published:** 2021-08-21

**Authors:** Caroline Espersen, Lise Borgwardt, Peter Nørgaard Larsen, Trine Borup Andersen, Louise Stenholt, Lars J. Petersen

**Affiliations:** 1 Department of Clinical Physiology, Nuclear Medicine and PET, Rigshospitalet, Blegdamsvej 9, 2100 Copenhagen, Denmark; 2 Department of Gastrointestinal Surgery, Rigshospitalet, Blegdamsvej 9, 2100 Copenhagen, Denmark; 3 Department of Nuclear Medicine, Aalborg University Hospital, Hobrovej 18-22, 9100 Aalborg, Denmark; 4 The Medical Library, Aalborg University Hospital, Sdr. Skovvej 15, 9000 Aalborg, Denmark; 5 Department of Clinical Medicine, University of Aalborg, Sdr. Skovvej 15, 9000 Aalborg, Denmark

**Keywords:** Nuclear imaging, Liver function, Liver volume, Future liver remnant, Preoperative assessment, Liver failure, Mortality

## Abstract

**Background/aims:**

Although volumetric imaging by computed tomography (CT) is the gold standard for preoperative assessment of the future liver remnant, nuclear imaging studies have shown promising data. This systematic review summarized the results from trials investigating volumetric and nuclear medicine imaging for the prediction of postoperative mortality and liver failure (LF).

**Methods:**

MEDLINE and Web of Science were searched for papers investigating nuclear imaging methods for the prediction of postoperative clinical outcomes in patients undergoing local, liver-directed treatments. Only papers investigating both preoperative nuclear imaging and CT or magnetic resonance imaging (MR) for the prediction of postoperative mortality and/or LF were included.

**Results:**

Twenty-five trials were qualified for this review. All trials but two used technetium-based tracers for the nuclear imaging examination. Four papers used MR imaging and the remaining used CT for the volumetric evaluation. Overall, the studies were heterogeneous both in terms of methodology and imaging technique. Of the thirteen studies reporting on postoperative mortality, most were descriptive without detailed diagnostic data. A few with detailed data found that nuclear imaging had better predictive value than volumetric imaging. Nineteen studies investigated the prediction of postoperative LF of which seven papers investigated the predictive value of both modalities in multivariable regression analysis. Two papers found that only nuclear imaging parameters were predictive of LF, one paper found that the CT parameter was predictive, and four papers found that combined nuclear and CT/MR imaging parameters were predictive of LF.

**Conclusion:**

Both methodologies were useful in the preoperative assessment of patients scheduled for liver interventions, especially in combination, but nuclear imaging demonstrated better predictive value for postoperative mortality and LF in a few trials. The overall technical and methodological heterogeneity of the included studies complicates the ability to directly compare the clinical utility of the two imaging techniques.

## Introduction

The volume of the future liver remnant (FLR) assessed by computed tomography (CT) is broadly considered the gold standard for preoperative liver assessment both in terms of determining eligibility for surgery and the extent of surgery [1]. By assuming a uniformly distributed liver function, CT-volumetry serves as an indirect assessment of the functional capacity of the FLR along with global liver function tests, biochemical markers, and clinical scores. However, in patients with underlying parenchymal liver disease, the functional capacity of the liver is not necessarily homogenously distributed. If the liver morphology and function are considered normal, 75–80% of the total liver volume can be safely resected [2–8]. However, if the liver function is suspected to be impaired, for example, due to chemotherapy or liver cirrhosis, the liver volume to be resected is greatly reduced [5, 7, 8].

With nuclear medicine imaging, it is possible to evaluate the function of the FLR directly. Therefore, one cutoff level for a safe liver resection might suffice irrespective of the underlying liver condition [9, 10]. Nuclear imaging may therefore offer a better risk estimation prior to liver surgery than volumetric assessments. Moreover, based on a previously published systematic review from the same literature search, preoperative nuclear imaging has been shown to predict postoperative liver failure (LF) in several studies [11]. Nonetheless, the incremental value of nuclear imaging techniques over volumetric imaging for preoperative assessment of the FLR has not yet been thoroughly clarified. The purpose of this systematic review was to summarize the studies investigating both preoperative functional imaging with nuclear medicine and volumetric imaging with CT or magnetic resonance (MR) imaging for the prediction of postoperative mortality and/or liver failure (LF) in patients undergoing local liver-directed treatments.

## Methods

### Literature search strategy

This review included papers from a previously published systematic review investigating preprocedural nuclear imaging methods for the prediction of postprocedural clinical outcomes after local intervention in the liver [11]. In brief, the literature search was performed in two bibliographic databases with a deadline of search of May 27, 2020. The criteria for patients, interventions, comparators, outcomes, and study design were customized for each bibliographic database [11]. The systematic review was conducted in accordance with the Preferred Reporting Items for Systematic Reviews and Meta-Analyses (PRISMA) guidelines [12].

### Eligibility criteria and study selection

The eligibility criteria have been described in detail previously [11]. For this sub-study of the original systematic review, we identified original studies where patients underwent a functional nuclear imaging-based examination as well as an anatomical examination by CT or MR imaging prior to localized, liver-directed treatments with the purpose of removing impaired liver tissue (e.g., liver resection, cryotherapy, radiotherapy, etc.). The included papers had to report the results of the preprocedural imaging examination with regard to  postprocedural clinical outcomes, including mortality and/or LF. For this analysis, we sought to investigate whether preprocedural nuclear imaging was significantly better than CT/MR imaging for the prediction of postprocedural mortality and LF.

### Data extraction

Two investigators reviewed relevant papers, and three investigators extracted data. We extracted details regarding study demographics, imaging technique and therapeutic interventions, and study methodology, such as patient enrollment (prospective/retrospective), selection (consecutive/non-consecutive), and outcome measures. Classification of major versus minor surgery was based on Couinaud’s criteria [13]. Extent of surgery (major vs. minor) was not included as a volumetric parameter for our data extraction, and only preoperative or combined pre-/perioperative volumetric measurements were included. Some studies analyzed mortality and LF as part of a composite outcome; such papers were included, denoted in the tables as ≠. Postoperative LF encompasses both terms described as posthepatectomy liver failure, hepatic insufficiency, liver dysfunction, and the like—in our paper, it is referred to as postoperative LF under one heading. Details on mortality and LF distinctions and data extraction have been described in further detail previously [11].

## Results

### Study demographics of the included papers

**Fig. 1:**
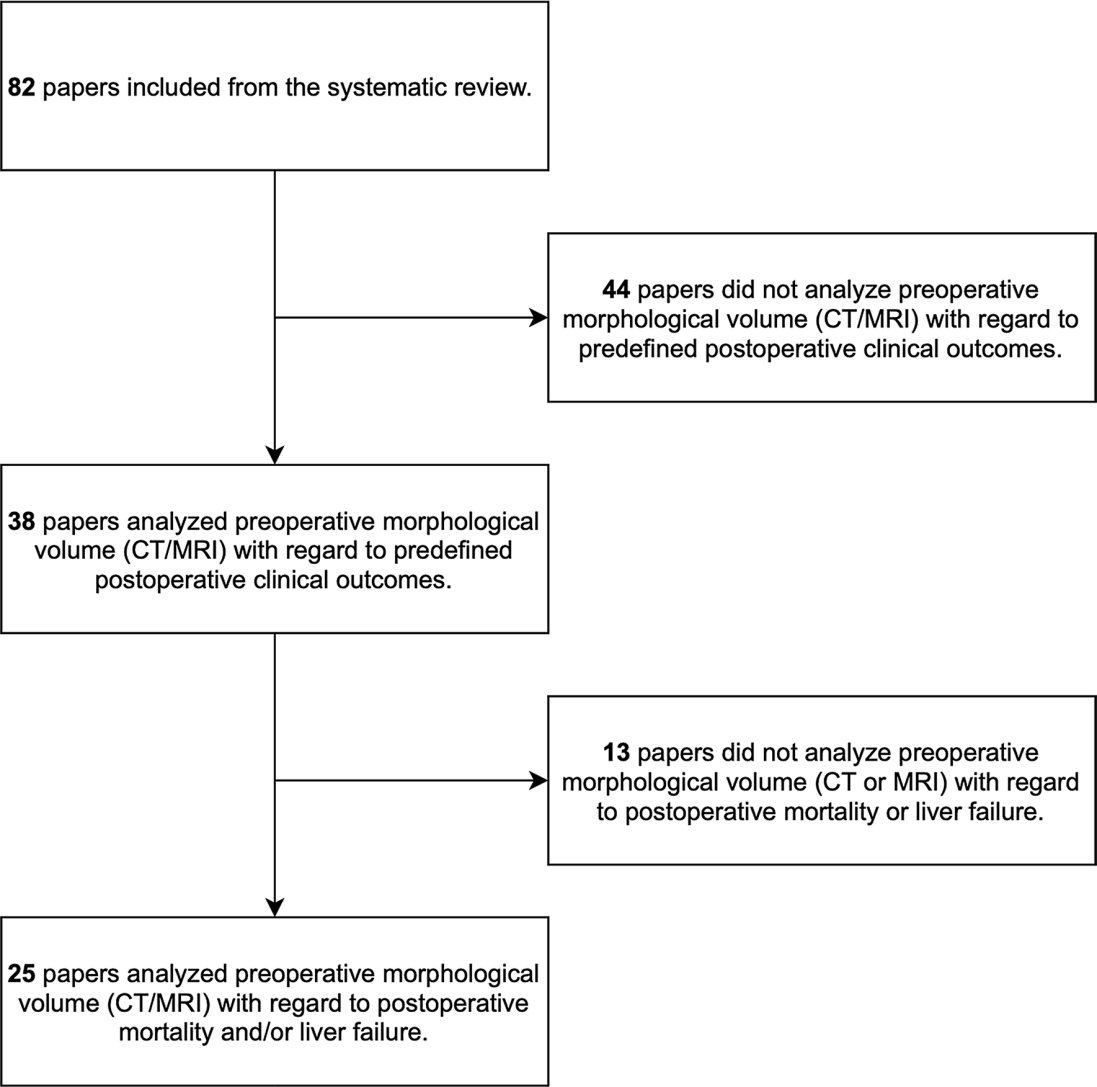
Consort diagram of the inclusion of papers for this review. CT: computed tomography, MRI: magnetic resonance imaging

From a total of 82 eligible studies included in the original systematic review, 25 papers investigated both preoperative nuclear medicine and volumetric imaging for the prediction of postoperative mortality and/or LF (Fig. 1). These 25 papers constituted the systematic review of functional versus anatomical preoperative imaging. Most trials were from Japan (*n* = 13), the Netherlands (*n* = 5), and Belgium (*n* = 3) (Table 1). The median publication year was 2013. Six studies enrolled patients prospectively. Consecutive recruitment was ensured in 20 trials. The median number of patients was 100 (range 11–625 patients). The surgical procedures involved both major and minor surgery. The nuclear imaging assessments commonly involved technetium-based tracers: [^99m^Tc]Tc-galactosyl human serum albumin (GSA) (*n* = 12) and [^99m^Tc]Tc-mebrofenin (*n* = 11). Two studies employed positron emission-based tracers [14, 15]. Most studies used CT for volumetric imaging; only four studies applied MR imaging. All studies used preoperative volumetric assessments; two papers investigated a combined pre-/peroperative volumetric parameter (resected parenchymal fraction) [16, 17]. Seven papers investigated a combined functional and volumetric preoperative parameter incorporating both volumetric and functional assessments. Overall, there were notable variations in the technical methodology, both in terms of the nuclear imaging parameters and calculations (also within the same tracer type) and the CT/MR imaging parameters (data not shown).

**Table 1:** Study demographics of the included papers reporting on preoperative functional nuclear medicine imaging and morphological volumetric imaging with computed tomography or magnetic resonance imaging for the prediction of mortality and/or liver failure

Author	Year	Country	Prospective study	Consecutive recruitment	Tracer	Patients (n)	Postoperative outcome		Major/minor surgery or both	Morphological volume assessment, CT/MRI	Preoperative, combined preoperative/peroperative volumetric measurement or both	Combined volumetric/ functional (NMI) parameter
							Mortality	Liver failure				
Chapelle et al. [18]	2016	Belgium	Yes	Yes	[^99m^Tc]Tc-Meb	88	Yes	Yes	Both	MRI	Preoperative	Yes
Chapelle et al. [27]	2017	Belgium	Yes	Yes	[^99m^Tc]Tc-Meb	188^a^	Yes	Yes	Both	MRI	Preoperative	Yes
Chapelle et al. [26]	2017	Belgium	No	Yes	[^99m^Tc]Tc-Meb	140	No	Yes	Both	MRI	Preoperative	Yes
Cho et al. [14]	2017	Korea	No	Yes	[^18^F]FDG	149	No	Yes	Both	CT	Preoperative	Yes
Cieslak et al. [21]	2016	The Netherlands	No	Yes	[^99m^Tc]Tc-Meb	163	Yes	Yes	Major	CT	Preoperative	No
de Graaf et al. [10]	2010	The Netherlands	No	Yes	[^99m^Tc]Tc-Meb	55	No	Yes	Major	CT	Preoperative	No
Dinant et al. [19]	2007	The Netherlands	Yes	Yes	[^99m^Tc]Tc-Meb	46	Yes	Yes	Both	CT	Preoperative	No
Fujioka et al. [33]	1999	Japan	No	No	[^99m^Tc]Tc-GSA	15	Yes	No	Both	CT	Preoperative	No
Hayashi et al. [20]	2015	Japan	No	Yes	[^99m^Tc]Tc-GSA	133	Yes	Yes	Major	CT	Preoperative	No
Hirai et al. [24]	2003	Japan	No	Yes	[^99m^Tc]Tc-GSA	30	No	Yes	Major	CT	Preoperative	No
Kokudo et al. [17]	2002	Japan	No	Yes	[^99m^Tc]Tc-GSA	111	No	Yes	Minor	CT	Combined	Yes
Kwon et al. [34]	2001	Japan	No	Yes	[^99m^Tc]Tc-GSA	47	Yes	No	Both^b^	CT	Preoperative	No
Kwon et al. [35]	2004	Japan	No	No	[^99m^Tc]Tc-GSA	32	Yes	No	Both	CT	Preoperative	No
Kwon et al. [36]	2006	Japan	No	Yes	[^99m^Tc]Tc-GSA	178	Yes	No	Both^b^	CT	Preoperative	No
Nanashima et al. [32]	2007	Japan	No	Yes	[^99m^Tc]Tc-GSA	185	No	Yes	Both	CT	Preoperative^c^	No
Nanashima et al. [37]	2013	Japan	No	No	[^99m^Tc]Tc-GSA	67	No	Yes	Both^b^	CT	Preoperative	No
Okabe et al. [30]	2014	Japan	Yes	Yes	[^99m^Tc]Tc-GSA	625	No	Yes	Both^b^	CT	Preoperative	No
Olthof et al. [22]	2017	The Netherlands	No	Yes	[^99m^Tc]Tc-Meb	116	Yes	Yes	Major	CT	Preoperative	No
Otsuki et al. [15]	1997	Japan	Yes	No	[^11^C]C-Met	11	Yes	No	Major	CT	Preoperative	Yes
Rassam et al. [23]	2019	The Netherlands	No	Yes	[^99m^Tc]Tc-Meb	71	Yes	Yes	Both	CT	Preoperative	No
Serenari et al.[6, 28]	2018	Argentina	No	Yes	[^99m^Tc]Tc-Meb	20	No	Yes	Major	CT or MRI	Preoperative	No
Serenari et al. [29]	2019	Italy	No	Yes	[^99m^Tc]Tc-Meb	27	No	Yes	Major	CT	Preoperative	No
Truant et al. [38]	2017	France	Yes	Yes	[^99m^Tc]Tc-Meb	100	Yes	No	Major	CT	Preoperative	No
Yoshida et al. [31]	2012	Japan	No	No	[^99m^Tc]Tc-GSA	256	No	Yes	Both	CT	Preoperative	Yes
Yumoto et al. [16]	2010	Japan	No	Yes	[^99m^Tc]Tc-GSA	101	No	Yes	Both	CT	Both	No

NMI: nuclear medicine imaging; CT: computed tomography; MRI: magnetic resonance imaging; [^99m^Tc]Tc-Meb: ^99m^Tc-labeled mebrofenin; [^99m^Tc]Tc-GSA: ^99m^Tc-labeled galactosyl human serum albumin; [^18^F]FDG: [^18^F]fluorodeoxyglucose; and [^11^C]C-Met: L-[methyl-^11^C]-methionine

^a^Including 88 patients from a prior study from Chapelle et al. [18]

^b^Both major and minor surgery but analyzed separately in the paper

^c^Based on the description in the paper, it is somewhat uncertain, whether the volumetric CT parameter was determined pre- or perioperatively. Here, we have assumed that the volumetric parameter was estimated with CT preoperatively

### Prediction of postoperative mortality

Thirteen papers reported on preoperative imaging for the prediction of postoperative mortality of which five were comparative studies with prespecified aims to compare functional, nuclear imaging with volumetric imaging for the prediction of postoperative mortality and/or LF [18–22] (Table 2). Most studies investigated 90-day mortality; mortality rates varied from 0 to 27%. Eight papers reported outcomes in a descriptive fashion and/or reported imaging parameters in patients with and without a fatal outcome without any statistical analyses. Four studies reported clinically relevant prognostic information for both imaging modalities. Two studies compared preoperative nuclear uptake values and CT volume measurements in patients with and without postoperative mortality [19, 23]. Dinant et al. found that uptake in the FLR determined by nuclear imaging was significantly lower in patients with LF-related mortality (mean 2.3 vs. 4.2%/min/BSA, *p* = 0.02), whereas volume of the FLR determined by CT was not significantly different in patients with and without LF-related mortality (mean 45 vs. 52%, *p* = 0.51) [19]. However, neither FLR uptake nor volume was significantly different in patients with and without overall mortality. Likewise, neither functional nor volumetric assessment of the FLR was significantly associated with 90-day mortality in patients with hepatocellular carcinoma undergoing liver resection [23].

**Table 2:** Overview of trials reporting on preoperative nuclear medicine imaging and computed tomography or magnetic resonance imaging for the prediction of postoperative mortality

Author	Mortality defined	Mortality event rate	Preset cutoff studied	Post hoc cutoff value established	Only descriptive analysis	Mortality versus no mortality comparison	Key diagnostic characteristics reported	CT/MRI or NMI predictive in univariable regression analysis	CT/MRI or NMI predictive in multivariable regression analysis	Comparison of predictive value
Dinant et al. [19]	No	11%	NMI/CT: No	NMI: Yes CT: No	No	NMI/CT: Yes	NMI/CT: Yes	NMI: Yes CT: NR	NMI: No CT: NR	NR
Chapelle et al. [18]	Yes	6%	NMI/MRI: No^a^	NMI/MRI: Yes^b^	Yes	NR	NR	NR	NR	NR
Chapelle et al. [27]	Yes	0%^c^	NMI/MRI: Yes^b^	NMI/MRI: No	Yes	NR	NR	NR	NR	NR
Cieslak et al. [21]	Yes	7%	NMI/CT: Yes^b^	NMI/CT: No	Yes	NR	NR	NR	NR	NR
Fujioka et al. ≠ [33]	No	7%	NMI/CT: No	NMI: Yes^b^ CT: No	Yes	NR	NR	NR	NR	NR
Hayashi et al. [20]	Yes	6%	NMI/CT: Yes^b^	NMI/CT: No	No	NMI/CT: NR	NMI/CT: NR	NMI: Yes CT: No	NMI/CT: NR	NR
Kwon et al. [34]	No	2%	NMI/CT: No	NMI/CT: Yes^b^	Yes	NR	NR	NR	NR	NR
Kwon et al. [35]	No	3%	NMI/CT: No	NMI/CT: Yes^b^	Yes	NR	NR	NR	NR	NR
Kwon et al. ≠ [36]	No	1%	NMI/CT: No	NMI: Yes^b^ CT: No	Yes	NR	NR	NR	NR	NR
Olthof et al. [22]	Yes	18%	NMI/CT: Yes^b^	NMI: Yes^b^ CT: No	No	NMI/CT: NR	NMI: Yes^b^ CT: Yes	NMI/CT: NR	NMI/CT: NR	NR
Otsuki et al. [15]	No	27%	NMI/CT: No	NMI: Yes^b^ CT: No	NMI: No CT: Yes	NMI: Yes CT: NR	NMI/CT: NR	NMI/CT: NR	NMI/CT: NR	NR
Rassam et al. [23]	Yes	11%	NMI: Yes^b^ CT: No	NMI/CT: No	No	NMI/CT: Yes	NMI/CT: NR	NMI/CT: NR	NMI/CT: NR	NR
Truant et al. [38]	No	14%^d^	NMI/CT: No	NMI/CT: No	Yes	NR	NR	NR	NR	NR

NMI: nuclear medicine imaging; CT: computed tomography; MRI: magnetic resonance imaging; and NR: not reported

^a^Only patients with a preoperative FLRV% > 25% were resected and included in the study

^b^The cutoff value is set for both avoiding mortality and postoperative LF or overall complications/poor postoperative outcome in order to obtain a safe hepatectomy

^c^No patients in the interventional group died postoperatively, but in the 88 patients in the prior study by Chapelle et al. the mortality rate was 6%, also reported in the table above

^d^Postoperative clinical outcomes were only reported in the seven patients who underwent ALPPS. One of the seven patients died due to LF postoperatively

In terms of the key diagnostic characteristics of the cutoff levels, Dinant et al. found that FLR function and volume had an area under the curve (AUC) of 88% (95%CI 75–100%) and 61% (95%CI 21–100%), respectively, for predicting LF-related mortality [19]. Olthof et al. found that FLR function and volume had an AUC of 70% (95%CI 57–82%) and 60% (95%CI 42–79%) for predicting posthepatectomy LF-related (PHLF) mortality [22]. None of the papers directly compared the predictive value of nuclear versus CT/MR imaging parameters for the prediction of postoperative mortality. In univariable logistic regression analysis, only marginal FLR function (OR 8.78, *p* = 0.008), and not FLR volume (OR 1.99, *p* = 0.367), was associated with an increased risk of postoperative mortality [20].

### Prediction of postoperative liver failure

Nineteen studies reported data for the prediction of postoperative LF of which seven were comparative studies with prespecified aims to compare functional, nuclear imaging with volumetric imaging for the prediction of postoperative LF [10, 18–22, 24] (Table 3). Most studies defined LF though the definitions varied across the studies (data not shown). The International Study Group of Liver Surgery’s criteria [25] for the definition of postoperative LF were used in several studies [18, 22, 23, 26–29]. The LF rate ranged from 1 to 33%. Seven papers investigated the predictive value of a predetermined cutoff value, eight papers established a post hoc cutoff value, and three studies investigated both [10, 22, 30]. Several studies (*n* = 15) reported detailed diagnostic data on the preoperative imaging tests for the prediction of postoperative LF (Table 4).

**Table 3:** Overview of trials reporting on preoperative nuclear medicine imaging and computed tomography or magnetic resonance imaging for the prediction of postoperative liver failure

Author	LF defined	LF event rate	Preset cutoff value studied	Post hoc cutoff value established	Only descriptive analysis	LF versus no LF comparison	Key diagnostic characteristics reported	Cutoff value predictive in univariable regression analysis	Cutoff value predictive in multivariable regression analysis
Chapelle et al. [18]	Yes	14%	NMI/MRI/Combined: No	NMI: No MRI/Combined: Yes^a^	No	NMI/MRI/Combined: Yes	NMI: No MRI/Combined: Yes	NMI: Yes MRI: Yes Combined: Yes	NMI: No MRI: No Combined: Yes
Chapelle et al. [27]	Yes	1%^b^	NMI: No MRI: Yes Combined: Yes	NMI/MRI/Combined: No	Yes	NR	NR	NR	NR
Chapelle et al. [26]	Yes	15%	NMI/MRI: No Combined: Yes	NMI/MRI/Combined: No	No	NMI/MRI/Combined: Yes	NMI/MRI/Combined: Yes	NMI: No MRI: Yes Combined: Yes	NMI: NR MRI: NR Combined: Yes
Cho et al. [14]	Yes	5%	NMI/CT/Combined: No	NMI/CT/Combined: Yes	No	NMI/CT/Combined: Yes	NMI/CT/Combined: Yes	NMI: No CT: Yes Combined: Yes	NMI: NR CT: No Combined: Yes
Cieslak et al. [21]	Yes	2%	NMI/CT: Yes	NMI/CT: No	Yes	NR	NR	NR	NR
de Graaf et al. [10]	Yes	16%	NMI: No CT: Yes	NMI: Yes CT: No	No	NMI/CT: Yes	NMI/CT: Yes	NMI/CT: NR	NMI/CT: NR
Dinant et al. [19]	Yes	13%	NMI/CT: No	NMI: Yes CT: No	No	NMI/CT: Yes	NMI/CT: Yes	NMI: Yes CT: NR	NMI: Yes CT: NR
Hayashi et al. [20]	Yes	8%	NMI/CT: Yes	NMI/CT: No	No	NMI/CT: NR	NMI/CT: NR	NMI: Yes CT: No	NMI/CT: NR
Hirai et al. [24]	Yes	23%	NMI/CT: No	NMI/CT: Yes	No	NMI/CT: Yes	NMI/CT: Yes	NMI/CT: NR	NMI/CT: NR
Kokudo et al. [17]	Yes^c^	13%	NMI/CT/Combined: No	NMI: No Combined: Yes CT: No	No	NMI/CT/Combined: Yes	NMI/CT/Combined: NR	NMI/CT/Combined: NR	NMI: No CT: NR Combined: Yes
Nanashima et al. ≠ [32]	Yes	6%	NMI/CT: No	NMI/CT: Yes	No	NMI/CT: Yes	NMI/CT: NR	NMI/CT: NR	NMI: No CT: Yes
Nanashima et al. [37]	No	4%	NMI: Yes CT: No	NMI/CT: No	Yes	NR	NR	NR	NR
Okabe et al. [30]	Yes	PLD: 7% LF: 2%	NMI: No CT: Yes	NMI: Yes (PLD) CT: No	No	NMI/CT: Yes (PLD)	NMI: Yes (PLD) CT: NR	NMI/CT: NR	NMI: Yes CT: NR (PLD)
Olthof et al. [22]	Yes	23%	NMI/CT: Yes	NMI/CT: Yes	No	NMI/CT: Yes	NMI/CT: Yes	NMI/CT: Yes	NMI: Yes CT: No
Rassam et al. ≠ [23]	Yes	10% (LF only)	NMI: Yes CT: No	NMI: No CT: No	No	NMI/CT: Yes	NMI/CT: NR	NMI/CT: NR	NMI/CT: NR
Serenari et al. [28]	Yes	20%^d^	NMI/CT/MRI: No	NMI: Yes CT/MRI: No	No	NMI/CT/MRI: Yes	NMI: Yes CT/MRI: NR	NMI/CT/MRI: NR	NMI/CT/MRI: NR
Serenari et al. [29]	Yes	33%	NMI: No CT: Yes	NMI/CT: No	NMI: No CT: Yes	NMI: Yes CT: NR	NMI/CT: NR	NMI/CT: NR	NMI/CT: NR
Yoshida et al. [31]	Yes	6%	NMI/CT/Combined: No	NMI/Combined: Yes CT: No	No	NMI/CT: Yes Combined: NR	NMI/Combined: Yes CT: NR	NMI/CT/Combined: NR	NMI: Yes CT: No Combined: NR
Yumoto et al. [16]	Yes^c^	17%	NMI/CT: No	NMI: Yes CT: No	No	NMI/CT: Yes	NMI: Yes CT: NR	NMI/CT: NR	NMI/CT: NR

LF: liver failure; NMI: nuclear medicine imaging; CT: computed tomography; MRI: magnetic resonance imaging; NR: not reported; and PLD: postoperative liver dysfunction

^a^Only patients with a preoperative FLRV% > 25% were resected and included in the study

^b^1% of patients in the interventional group developed LF postoperatively, but in the 88 patients in the prior study by Chapelle et al. the liver failure rate was 14%, also reported in the table above

^c^Signs of postoperative LF

^d^Including four patients who developed LF after stage 2 of the ALPPS procedure and excluding one patient who developed LF after stage 1 of the ALPPS procedure

**Table 4:** Details of the diagnostic characteristics and predictive values of preoperative nuclear medicine imaging and computed tomography or magnetic resonance imaging for predicting postoperative liver failure (both combined functional/morphological parameters and separate morphological and functional parameters)

Author	Cutoff value (variable: value and unit)	LF versus no LF (mean or median, p-value)	Key diagnostic characteristics	Univariable regression analysis (impact, p-value)	Multivariable regression analysis (impact, p-value)	Significant differences in key diagnostic characteristics between NMI and CT/MRI
Chapelle et al. [18]	NMI: TLF: NR (%/min)^a^	5.0 versus 6.2 (*p* = 0.020)	NR	NR (*p* = 0.027)	NR (NS)	NR
	CT: FLRV%: 40 (%)	49 versus 76 (*p* < 0.001)	Sens 71%, spec 91%, PPV 41%, NPV 97%, AUC 0.77	For FLRV% < 40: OR 26 (*p* < 0.001)	NR (NS)	
	NMI/MRI combined: eFLRF: 2.3 (%/min/m^2^)	2.2 versus 4.7 (*p* < 0.001)	Sens 92%, spec 98%, PPV 92%, NPV 99%, AUC 0.89	For eFLRF < 2.3: OR 836 (*p* < 0.001)	NR (*p* = 0.001)	
Chapelle et al. [26]	NMI: HBS^BSA^: NR (%/min)^a^	5.5 versus 6.1 (NS)	AUC 0.652	NR (NS)	NR (NR)	NR
	CT: FLRV%: NR (%)	60.6 versus 80.9 (*p* < 0.001)	AUC 0.800	NR (*p* < 0.001)	NR (NR)	
	NMI/MRI combined: eFLRF: 2.3 (%/min/m^2^)	3.3 versus 8.4 (*p* < 0.001)	AUC 0.843	NR (*p* < 0.001)	OR 0.35 (*p* = 0.002)	
Cho et al. [14]	NMI: SUV_mean_: 2.4	2.1 versus 2.3 (NS)	Sens 100%, spec 32%, PPV 7%, NPV 100%	For SUV_mean_ ≤ 2.4: OR 7.0 (NS)	NR (NR)	NR
	CT: Predicted remnant hepatic volume: 415.8 (cm^3^)	488.5 versus 652.3 (NS)	Sens 71%, spec 81%, PPV 16%, NPV 98%	For predicted remnant hepatic volume ≤ 415.8: OR 10.6 (*p* = 0.006)	NR (NS)	
	CT: RFRHV: 0.3	0.4 versus 0.5 (*p* = 0.007)	Sens 71%, spec 88%, PPV 23%, NPV 98%	For RFRHV ≤ 0.3: OR 18.4 (*p* = 0.001)	NR (NS)	
	NMI/CT combined: TLG_r_: 625.6	1067 versus 1491 (NS)	Sens 57%, spec 97%, PPV 44%, NPV 98%	For TLG_r_ ≤ 625.6: OR 36.5 (*p* < 0.001)	For TLG_r_ ≤ 625.6: OR 82.9 (*p* < 0.001)	
de Graaf et al. [10]	NMI: FRL-F: 2.69 (%/min/m^2^)	2.2 versus 4.3 (*p* = 0.001)	Sens 89%, spec 87%, PPV 57%, NPV 98%, AUC 0.92	NR (NR)	NR (NR)	NR
	CT: %FRL-V: < 30 (normal liver), < 40 (compromised liver) (%)	35.0 versus 49.7 (*p* = 0.013)	Sens 78%, spec 80%, PPV 44%, NPV 95%	NR (NR)	NR (NR)	
	CT: sFRL: < 30 (normal liver), < 40 (compromised liver) (%)	35.2 versus 49.2 (*p* = 0.018)	Sens 67%, spec 87%, PPV 50%, NPV 93%	NR (NR)	NR (NR)	
Dinant et al. [19]	NMI: FRL-uptake: 2.5 (%/min/BSA)	2.3 versus 4.3 (*p* = 0.003)	Sens 83%, spec 90%, PPV 56%, NPV 97%, AUC 0.90	NR (*p* = 0.01)	OR 4.0 (*p* = 0.03)	NR
	CT: FRL-volume: NR (%)	42 versus 52 (NS)	AUC 0.65	NR (NR)	NR (NR)	
Hayashi et al. [20]	NMI: Marginal FR function: depending on ICG-value (see paper for further information) (%)	NR (NR)	NR	For marginal FR function versus safe FR function: OR 11.0 (*p* = 0.001)	NR (NR)	NR
	CT: Marginal FR volume: depending on ICG-value (see paper for further information) (%)	NR (NR)	NR	For marginal FR volume versus safe FR volume: OR 2.3 (NS)	NR (NR)	
Hirai et al. [24]	NMI: [^99m^Tc]Tc-GSA uptake in the FLR: 25 (%)	Before PVE: 10.0 versus 25.8 (*p* = 0.02) After PVE: 18.0 versus 38.4 (*p* = 0.01)	Sens 50%, spec 94%, AUC 0.97^b^	NR (NR)	NR (NR)	No
	CT: Ratio of FLR volume to standard liver volume: 35 (%)	Before PVE: 33.0 versus 38.5 (NS) After PVE: 33.7 versus 46.2 (*p* = 0.003)	Sens 57%, spec 91%, AUC 0.93^b^	NR (NR)	NR (NR)	
Kokudo et al. [17]^c^	NMI: LHL15: NR	0.89 versus 0.93 (*p* = 0.025)	NR	NR (NR)	NR (NS)	NR
	NMI: HH15: NR	0.58 versus 0.52 (NS)	NR	NR (NR)	NR (NR)	
	NMI: R_0_: NR (μmole)	0.14 versus 0.18 (*p* = 0.038)	NR	NR (NR)	NR (NS)	
	NMI: [R]_0_: NR (μM)	0.63 versus 0.70 (NS)	NR	NR (NR)	NR (NR)	
	CT: RPF: NR (%)	32.5 versus 27.1 (NS)	NR	NR (NR)	NR (NR)	
	Combined NMI/CT: R_0_-remnant: 0.16 (μmole)	0.015 versus 0.024 (*p* = 0.011)	NR	NR (NR)	Per 0.01 μmole increment: HR 0.82 (*p* = 0.022)	
Nanashima et al. ≠ [32]	NMI: LHL15: 0.85	91.1 versus 93.1 (*p* = 0.014)	NR	NR (NR)	For LHL15 < 0.85 versus ≥ 0.85: OR 1.4 (NS)	NR
	CT: Volume of resected liver: 50 (%)	45 versus 27 (*p* < 0.01)	NR	NR (NR)	For volume of resected liver ≥ 50 versus < 50%: OR 7.0 (*p* = 0.027)	
Okabe et al. [30]	NMI: LHL15: 0.93^d^	0.92 versus 0.93 (*p* = 0.0027)	Sens: 88%, spec 96%	NR (NR)	For LHL15 ≤ 0.93: OR 7.4 (*p* = 0.0082)	NR
	NMI: HH15: NR^d^	0.66 versus 0.58 (*p* = 0.0041)	NR	NR (NR)	NR (NS)	
	CT: %FLR: NR (%)^d^	50.3 versus 50.6 (NS)	NR	NR (NR)	NR (NR)	
Olthof et al. [22]	NMI: Total liver function: NR (%/min)	14.6 versus 16.2 (*p* = 0.41)	NR	NR (NR)	NR (NR)	NR
	NMI: FLR function: NR (%)	44.7 versus 63.4 (*p* < 0.01)	AUC: 0.68	NR (NR)	NR (NR)	
	NMI: FLR function: 8.5 (%/min) (post hoc cutoff)	5.6 versus 8.7 (*p* < 0.01)	PPV 36%, NPV 91% ^e^, AUC 0.69	For FLR function < 8.5: OR 5.4 (*p* < 0.01)	For FLR function < 8.5: OR 4.1 (*p* < 0.01)	
	NMI: sFLR function: 2.7 (%/min/m^2^) (predefined cutoff)	3.1 versus 4.7 (*p* < 0.01)	PPV 38%, NPV 82% ^e^, AUC 0.68	NR (NR)	NR (NR)	
	CT: FLRV%: 30 (%) (predetermined cutoff), 28.7% (post hoc cutoff)	43 versus 54 (NS)	Predetermined cutoff: PPV 56%, NPV 83%, AUC 0.60 Post hoc cutoff: PPV 71%, NPV 83%	For FLRV% < 30: OR 5.2 (*p* < 0.01)	For FLRV% < 30: OR 3.4 (NS)	
	CT: sFLRV%: NR (%/m^2^)	NR (NR)	AUC 0.58	NR (NR)	NR (NR)	
	CT: FLRV: NR (mL)	760 versus 986 (NS)	NR	NR (NR)	NR (NR)	
	CT: Total liver volume: NR (mL)	2016 versus 1841 (NS)	NR	NR (NR)	NR (NR)	
Rassam et al. ≠ [23]	NMI: FRLF: 2.7 (%/min/m^2^) (predefined cutoff)	NR (NS)	NR	NR (NR)	NR (NR)	NR
	NMI: MUR: NR (%/min)	NR (NS)	NR	NR (NR)	NR (NR)	
	CT: FRLV%: NR (%)	NR (NS)	NR	NR (NR)	NR (NR)	
Serenari et al. [28]	NMI: FLR-C: 34.5 (%)	30 versus 41 (*p* = 0.011)	Sens 100%, spec 82%, PPV 50%, NPV 100%	NR (NR)	NR (NR)	NR
	NMI: FLR-F: 1.69 (%/min/m^2^)	0.94 versus 2.07 (*p* = 0.011)	Sens 100%, spec 75%, PPV 50%, NPV 100%	NR (NR)	NR (NR)	
	NMI: HIBA-i: 14.94 (%)	12.86 versus 23.29 (*p* = 0.001)	Sens 100%, spec 94%, PPV 80%, NPV 100%	NR (NR)	NR (NR)	
	CT: FLR/sTLV: NR (%)	35 versus 42 (NS)	NR	NR (NR)	NR (NR)	
	CT: FLR/mTLV: NR (%)	34 versus 41 (NS)	NR	NR (NR)	NR (NR)	
	CT: FLR/BW: NR (%)	0.74 versus 0.88 (NS)	NR	NR (NR)	NR (NR)	
Serenari et al. [29]	NMI: FLR-F: NR (%/min/m^2^)	1.72 versus 4.02 (NR)	NR	NR (NR)	NR (NR)	NR
Yoshida et al. [31]	NMI: rLUV_(BSA)_: 27.0	23.0 versus 33.6 (*p* < 0.001)^f^	Sens 91%, spec 81%, PPV 31%, NPV 99%, AUC 0.89	NR (NR)	NR (*p* < 0.001)	NR
	NMI: rLUV_(BW)_: 0.66	NR (NR)	Sens 80%, spec 84%, PPV 24%, NPV 99%, AUC 0.85	NR (NR)	NR (NR)	
	NMI: rLUR: 50.0 (%)	NR (NR)	Sens 93%, spec 66%, PPV 15%, NPV 99%, AUC 0.87	NR (NR)	NR (NR)	
	NMI: HH15: NR	0.64 versus 0.60 (*p* < 0.05)^f^	NR	NR (NR)	NR (NS)	
	NMI: LHL15: NR	0.90 versus 0.91 (NS)^f^	NR	NR (NR)	NR (NR)	
	NMI: %remnant LF: NR (%)	60.9 versus 75.3 (*p* < 0.001)^f^	NR	NR (NR)	NR (NS)	
	CT: % remnant LV: NR (%)	61.5 versus 73.7 (*p* < 0.05)^f^	NR	NR (NR)	NR (NS)	
	Combined NMI/CT: rLUV_(LV)_: 0.21	NR (NR)	Sens 92%, spec 46%, PPV 10%, NPV 99%, AUC 0.73	NR (NR)	NR (NR)	
Yumoto et al. [16]	NMI: R0-remnant: 100 (nmol/liver)	62.1 versus 122.2 (*p* < 0.001)	AUC: 0.97	NR (NR)	NR (NR)	NR
	NMI: [R]0: NR (nmol/l)	412 versus 551 (*p* = 0.045)	AUC: 0.80	NR (NR)	NR (NR)	
	NMI: R0: NR (nmol/liver)	149.8 versus 211.2 (*p* = 0.047)	NR	NR (NR)	NR (NR)	
	NMI: LHL15: NR	0.79 versus 0.87 (*p* = 0.035)	AUC: 0.74	NR (NR)	NR (NR)	
	CT: TLV: NR (mL)	1684.3 versus 1429.7 (NS)	NR	NR (NR)	NR (NR)	
	CT: Remnant liver volume: NR (mL)	741.7 versus 854.0 (NS)	NR	NR (NR)	NR (NR)	
	CT: RPF: NR (%)	36.9 versus 31.7 (*p* = 0.046)	NR	NR (NR)	NR (NR)	

LF: liver failure; NMI: nuclear medicine imaging; CT: computed tomography; MRI: magnetic resonance imaging; NR: not reported; NS: not significant; OR: odds ratio; AUC: area under the curve; HR: hazard ratio; Sens: sensitivity; Spec: specificity; PPV: positive predictive value; NPV: negative predictive value; TLF: total liver function; FLRV%: the future liver remnant volume as a percentage of total liver volume; eFLRF: estimated future liver remnant function; HBS^BSA^: global liver function ([^99m^Tc]Tc-mebrofenin hepatobiliary scintigraphy clearance divided by body surface area); SUV_mean_: mean standardized uptake value; RFRHV: ratio of remnant hepatic volume to preoperative hepatic volume; TLG_r_: total glycolysis of the remnant liver; FRL-F: future remnant liver uptake function; %FRL-V: future remnant liver volume as a percentage of total liver volume; sFRL: standardized future remnant liver; FRL: future remnant liver; FR: future remnant; FLR: future liver remnant; PVE: portal vein embolization; LHL15: [^99m^Tc]Tc-GSA receptor index; HH15: [^99m^Tc]Tc-GSA clearance index; R_0_: total hepatic asialoglycoprotein receptor amount; [R]_0_: hepatic asialoglycoprotein receptor concentration; RPF: resected parenchymal fraction; R_0_-remnant: total hepatic asialoglycoprotein receptor amount in the future remnant liver; %FRL: future remnant liver volume as a percentage of total liver volume; sFLRV%: standardized future liver remnant volume ratio; FRLF: future remnant liver function; FRLV: future remnant liver volume; MUR: Mebrofenin uptake rate; FLR-C: percentage of counts within the future remnant liver; HIBA-i: the HIBA-index (the proportion of radionuclide accumulated in the future remnant liver); FLR/sTLV: the ratio between future liver remnant volume and standardized total liver volume; FLR/mTLV: the ratio between future liver remnant volume and measured total liver volume; FLR/BW: the ratio between future liver remnant volume and body weight; rLUV_(BSA)_: liver uptake value of the remnant liver corrected for body surface area; rLUR: remnant liver uptake ratio; LUV_(BW)_: liver uptake value of the remnant liver corrected for body weight; %remnant LF: the relative residual liver function; % remnant LV: the relative residual liver volume; rLUV_(LV)_: liver uptake value of the remnant liver corrected for liver volume; and TLV: total liver volume

For the diagnostic characteristics, sensitivity, specificity, negative predictive value, positive predictive value, and area under the curve were reported if available

^a^TLF and HBS^BSA^ correspond to the same global liver function estimate from [^99m^Tc]Tc-mebrofenin hepatobiliary scintigraphy, but in the two papers they are named differently

^b^After PVE

^c^For “signs of postoperative liver failure”

^d^Reported here for patients with a %FLR (volume-based) of 35–60%, further details are provided for patients > 60% FLR in the original paper

^e^In the whole patient population, for information on characteristics of NMI for patients with bilirubin level < 50 μmol/L at the time of the hepatobiliary scintigraphy, see the paper

^f^Non-preserved versus preserved liver function on postoperative day 5; non-preserved referring to moderate–severe hepatic dysfunction on postoperative day 5; and preserved referring to no or mild hepatic dysfunction on postoperative day 5

Fourteen studies compared the results of both preoperative nuclear and CT/MR imaging in patients with and without postoperative LF. Eleven of these papers demonstrated a significantly lower functional liver capacity based on preoperative nuclear imaging in patients with postoperative LF compared to those without. In contrast, eight of these papers reported a significantly lower volumetric liver capacity or higher resected liver volume based on preoperative CT/MR imaging in patients with postoperative LF compared to those without. Four studies investigated the results of a combined nuclear and CT/MR imaging parameter in patients with and without LF [14, 17, 18, 26], of which three found a statistically significant difference.

Overall, the sensitivity and specificity of the nuclear imaging cutoffs for the prediction of postoperative LF varied across the studies from 50 to 100% and 32 to 96%, respectively. In comparison, the specificity and sensitivity of the CT/MR imaging cutoff varied from 57 to 78% and 80 to 91%, respectively. Lastly, the combined nuclear and CT/MR imaging parameters resulted in sensitivities and specificities ranging from 57 to 92% and 46 to 98%, respectively. The volumetric parameter (FLRV%) and the combined nuclear and MR imaging parameter (eFLRF) had a higher AUC than the nuclear imaging parameter alone (HBS^BSA^) for the prediction of postoperative LF [26]. Moreover, nuclear imaging with [^99m^Tc]Tc-GSA was not significantly better in predicting LF than CT-volumetry when comparing the AUC for the prediction of LF for the nuclear (AUC: 0.97) and CT (AUC: 0.93) imaging parameter [24]. Cho et al. found that the volumetric parameter had a higher AUC for the prediction of postoperative LF, although not significantly better than that of the other evaluated parameters including nuclear functional imaging [14].

Five papers analyzed the predictive value of both a nuclear and CT/MR imaging parameter for the prediction of postoperative LF in univariable regression analyses [14, 18, 20, 22, 26]. Two of these found that both the nuclear and CT/MR imaging parameters were predictive of LF [18, 22]. However, whereas Hayashi et al. only demonstrated a significant predictive value of marginal future remnant function but not volume [20], two papers demonstrated a significant predictive value of the CT/MR imaging parameter alone and not the nuclear imaging parameter in univariable regression analyses [14, 26]. Moreover, three papers found that a combined nuclear and volumetric imaging parameter was predictive of LF in univariable regression analyses [14, 18, 26]. Seven papers investigated the predictive value of both nuclear and CT/MR imaging parameters or a combination of both in multivariable regression analysis. Two papers demonstrated a significant predictive value of the nuclear and not CT imaging parameter for the prediction of postoperative LF [22, 31], whereas Nanashima et al. only demonstrated a significant predictive value of the estimated resected volume (%) from the CT examination for overall postoperative complications including LF in multivariable analysis [32]. Four papers analyzed the predictive value of a combined functional and morphological parameter (combined nuclear and CT/MR imaging) [14, 17, 18, 26]. These papers found that the combined parameters were predictive of LF in both univariable and/or multivariable analysis. Three of these papers also investigated strictly nuclear and/or the CT/MR imaging parameters and found that they were not predictive of postoperative LF in multivariable analysis [14, 17, 26].

In terms of historical comparisons, mortality and LF rates were significantly higher in an observational study using only FLR volume ratio for determining eligibility for hepatectomy compared to a prospective interventional study employing the FLR function cutoff for determining eligibility (6 vs. 0%, *p* = 0.016 and 15 vs. 14%, *p* = 0.001, respectively) [27].

## Discussion

Several studies have investigated both preoperative nuclear and CT/MR imaging for predicting postoperative clinical outcomes in the same study setup. Both functional nuclear imaging and volumetric imaging with CT/MR were useful in the preoperative assessment of patients undergoing liver surgery, especially in combination. However, nuclear imaging demonstrated a better predictive value for postoperative mortality and LF than CT/MR imaging alone in a few studies. Yet, the methodology, imaging techniques, and parameters across the studies were heterogeneous and detailed diagnostic data were limited, especially in terms of postoperative mortality. Only a few of the trials included prespecified aims to compare preoperative nuclear imaging with volumetric imaging for the prediction of postoperative mortality and/or LF. As a result, despite theoretical advantages of nuclear imaging, it is difficult to directly compare the clinical utility of the two imaging techniques across the included studies and determine whether nuclear functional imaging offers incremental value as opposed to CT/MR volumetric imaging for the prediction of both postoperative mortality and LF.

Our review of the published reports revealed very limited detailed diagnostic data on the association between preoperative nuclear and CT/MR imaging and postoperative mortality. Among the few studies with detailed diagnostic data, the AUC for predicting LF-related mortality was higher for the nuclear imaging techniques compared to CT, although no statistical comparison of difference in the AUC was conducted. Moreover, one study found that the preoperative FLR functional uptake and not FLR volume was significantly lower in patients with postoperative LF-related mortality compared to those without [19], whereas neither function nor volume of the FLR was associated with overall mortality. Only one paper [20] investigated the predictive value of both CT and nuclear imaging in univariable analysis and found that only marginal FRL function and not marginal FRL volume was predictive of postoperative mortality.

There were several studies with detailed diagnostic data on the predictive value of preoperative nuclear and CT/MR imaging parameters with regard to postoperative LF. Overall, more papers demonstrated significant differences in preoperative nuclear imaging uptake values in patients with and without postoperative LF as opposed to differences in the preoperative CT imaging parameters (11 vs. 8 papers). Moreover, nuclear imaging more often proved to be an independent significant predictor of postoperative LF in multivariable analyses compared to CT/MR imaging (in 2 vs. 1 paper). However, the differences were too small for firm conclusions about the predictive value of nuclear vs. CT/MR imaging. Moreover, two studies compared the predictive value of the nuclear versus CT imaging parameters directly and found no significant differences in the AUC of the nuclear and CT imaging technique for predicting postoperative LF [14, 24]. However, in all but one of the comparative studies with prespecified aims to compare nuclear imaging with volumetric imaging for the prediction of postoperative LF, nuclear imaging, or combined nuclear/volumetric imaging proved to be better than volumetric imaging alone for predicting postoperative LF [10, 18–22]. These studies highlight the theoretical and clinical importance of functional nuclear imaging in the preoperative assessment.

Interestingly, when combining the preoperative functional and volumetric imaging results in a single parameter, the combined parameter demonstrated optimized predictive value for estimating the risk of postoperative LF [14, 17, 18, 26]; the combined parameter showed better predictive potential for postoperative LF than did nuclear or CT/MR imaging alone. These findings underscore the importance of both volumetric and functional assessments of the FLR and the added potential of incorporating both in the preoperative assessment.

Overall, there was a great heterogeneity in the included studies both in terms of methodology, aims, study populations as well as the nuclear imaging acquisition techniques and calculation of the nuclear and CT/MR imaging parameters. In a minority of studies, the prespecified primary or secondary aims involved comparing functional nuclear imaging with volumetric imaging for predicting postoperative mortality and/or LF [10, 18–22, 24]. Other studies evaluated several pre- or perioperative factors associated with postoperative outcomes including nuclear and volumetric imaging [30, 32]. In most studies, however, the volumetric evaluation with regard to postoperative mortality and/or LF was part of additional results not necessarily pertaining to the main objectives of the study. Moreover, the study populations varied both in terms of the number of patients included, the underlying indication for liver surgery, and the involved surgical technique (major vs. minor surgery, and staged hepatectomy). Moreover, the small samples sizes of some of the original studies may cause selection bias due to the effective use of functional imaging in these patient groups. The methodological and technical differences in the studies complicate the comparison of functional versus volumetric imaging across the studies and warrant a standardization of methodology and imaging techniques. This would offer a better comparison of nuclear imaging versus CT/MR imaging in the preoperative assessment of postoperative risk in patients undergoing liver surgery.

Whereas volumetric imaging with CT is a well-established routine examination prior to liver surgery for evaluating resectability and eligibility for surgery, there are still no widely accepted international guidelines or consensus statements on the use of nuclear imaging in the preoperative assessment. Nonetheless, nuclear imaging has gained increasing use in the preoperative assessment over the last decade. The limitations of volumetric imaging as an indirect estimate of the functional capacity of the FLR seem to be overcome by the potential of nuclear imaging to directly estimate the regional function of FLR and thus the actual postoperative functional capacity. Indeed, our previously published systematic review focusing solely on the predictive value of nuclear imaging techniques for the prediction of postoperative mortality and LF revealed that despite technical and methodological heterogeneity across the studies, a notable number of trials found a significant predictive value of nuclear imaging for the prediction of postoperative LF.

As this review is based on studies included in a previously published systematic review of nuclear medicine imaging methods [11], the literature search was set up to find original research papers investigating preoperative nuclear imaging for the prediction of postoperative clinical outcomes and not necessarily papers comparing nuclear to CT/MR imaging for the prediction of postoperative clinical outcomes which was the focus of this review. Therefore, this analysis should not be regarded as a systematic review of anatomical imaging methods: There are likely many trials examining anatomical methods without comparison to nuclear medicine methods. The literature search for original papers covered papers published up until May 27, 2020, so we may have missed very recent reports. Moreover, some groups published several similarly appearing papers with increasing number of included patients and we did not contact individual authors to enquire about overlapping data. This may, therefore, cause a potential bias due to overlapping data.

## Conclusion

In conclusion, 25 papers investigated both preoperative nuclear imaging and CT/MR imaging for predicting postoperative clinical outcomes in patients undergoing local, liver-directed treatments. Both volumetric imaging and nuclear imaging were useful in the preoperative assessment of postoperative risk, especially in combination, but nuclear imaging demonstrated a better predictive value for postoperative mortality and LF than volumetric imaging alone in a few trials. However, the abundant technical and methodological heterogeneity across the included studies complicates the ability to directly compare the results of functional nuclear imaging with that of volumetric imaging with CT/MR for the prediction of postoperative mortality and LF. Larger, prospective studies would be beneficial in order to establish the added benefit of nuclear imaging to the standard of care, CT/MR imaging, in the preoperative assessment.

## Data Availability

All data generated or analyzed in this study are included in this published article.

## Abbreviations

FLR: Future liver remnant
CT: Computed tomography
MR: Magnetic resonance
LF: Liver failure
GSA: (Human) galactosyl serum albumin
AUC: Area under the curve
